# Alternatives to Outdoor Daylight Illumination for Photodynamic Therapy—Use of Greenhouses and Artificial Light Sources

**DOI:** 10.3390/ijms17030309

**Published:** 2016-02-29

**Authors:** Catharina M. Lerche, Ida M. Heerfordt, Jakob Heydenreich, Hans Christian Wulf

**Affiliations:** Department of Dermatology, Bispebjerg Hospital, University of Copenhagen, Bispebjerg Bakke 23, DK-2400 Copenhagen, Denmark; ida.marie.heerfordt.02@regionh.dk (I.M.H.); Jakob.heydenreich@regionh.dk (J.H.); h.wulf@regionh.dk (H.C.W.)

**Keywords:** artificial daylight, photodynamic therapy, greenhouse, daylight PDT, methyl aminolevulinate

## Abstract

Daylight-mediated photodynamic therapy (daylight PDT) is a simple and pain free treatment of actinic keratoses. Weather conditions may not always allow daylight PDT outdoors. We compared the spectrum of five different lamp candidates for indoor “daylight PDT” and investigated their ability to photobleach protoporphyrin IX (PpIX). Furthermore, we measured the amount of PpIX activating daylight available in a glass greenhouse, which can be an alternative when it is uncomfortable for patients to be outdoors. The lamps investigated were: halogen lamps (overhead and slide projector), white light-emitting diode (LED) lamp, red LED panel and lamps used for conventional PDT. Four of the five light sources were able to photobleach PpIX completely. For halogen light and the red LED lamp, 5000 lux could photobleach PpIX whereas 12,000 lux were needed for the white LED lamp. Furthermore, the greenhouse was suitable for daylight PDT since the effect of solar light is lowered only by 25%. In conclusion, we found four of the five light sources and the greenhouse usable for indoor daylight PDT. The greenhouse is beneficial when the weather outside is rainy or windy. Only insignificant ultraviolet B radiation (UVB) radiation passes through the greenhouse glass, so sun protection is not needed.

## 1. Introduction

**Figure 1:**
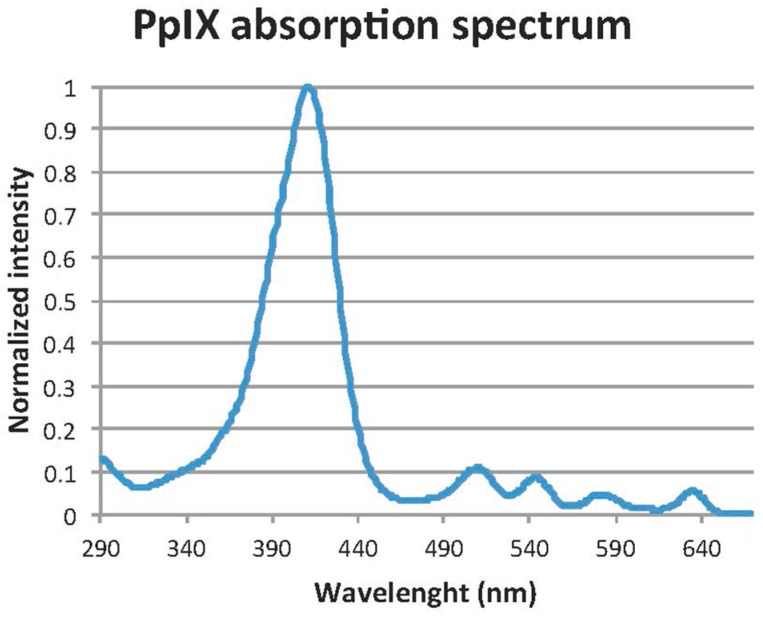
Photobleach protoporphyrin IX (PpIX) absorption spectrum. Reproduced with permission from Stine R. Wiegell, British Journal of Dermatology; published by Wiley and Sons, 2009.

Daylight photodynamic therapy (daylight-PDT) has become an established treatment for actinic keratoses [1]. The daylight PDT regimen includes superficial curettage, application of methyl aminolevulinate (MAL) and two hours outdoors in daylight starting 30 min. after MAL application [2,3]. During daylight PDT the photosensitizer protoporphyrin IX (PpIX) is produced and photobleached continuously resulting in much less pain than conventional PDT and an excellent cosmetic outcome [2,4,5,6]. However, the treatment is very dependent on geographical location (latitude), weather conditions and time of year [3,7]. In Denmark it is only possible to perform daylight-mediated PDT from April to November due to low temperatures and insufficient daylight doses the rest of the year [3,7]. Treatment rooms with artificial “daylight” would enable us to treat patients all year round, also on rainy days during summer. In the infancy of PDT, slide projectors were used as a light source before LED technology became easily available [8,9]. Attempts have been made to develop other artificial daylight illumination systems, but these are not yet commercially available [10]. All PpIX absorption peaks are within the visible spectrum of light (Figure 1) [11]. Therefore, a range of light sources can be used to photobleach PpIX [1]. Besides artificial “daylight” sources, it might be possible to use a greenhouse in rainy, cold or windy weather conditions with sufficient natural daylight.

Therefore, the present study investigated lamps already on the market with the aim of clarifying which are the most suitable for indoor PDT with continuous “daylight” illumination. We measured the spectrum of different artificial “daylight” sources and calculated the fluence rate and PpIX light fluence received within two hours of exposure for different distances from the lamp. We also determined the amount of daylight available in a glass greenhouse situated outdoors in the open. We measured the spectrum and calculated the fluence rate and PpIX light fluence inside and outside the greenhouse for two different weather conditions: clear blue sky and heavily overcast. We then investigated at what visible light intensity (measured in lux) the lamps and greenhouse were able to photobleach PpIX in persons with healthy skin.

## 2. Results and Discussion

Using a spectroradiometer we measured the spectrum of different artificial light sources. In addition, we measured the daylight spectrum inside/outside the greenhouse under different weather conditions. To be able to calculate the PpIX light fluence rate (mW/cm^2^ PpIX weighted) the spectral measurements were weighted with the normalized (412 nm) absorption spectrum for PpIX shown in Figure 1 [11]. We added a measurement of the illuminance of all light sources using a lux meter since it is much easier to use a lux meter than a spectroradiometer.

It is known that there is a linear dependence between increasing PpIX light dose and increasing response rate for actinic keratosis [1]. Although the optimal fluence for daylight PDT is thought to be above 8 J/cm^2^, as low as 3.5 J/cm^2^ has also proved to be sufficient [1].

### 2.1. Results from Five Different Artificial Daylight Sources

**Figure 2:**
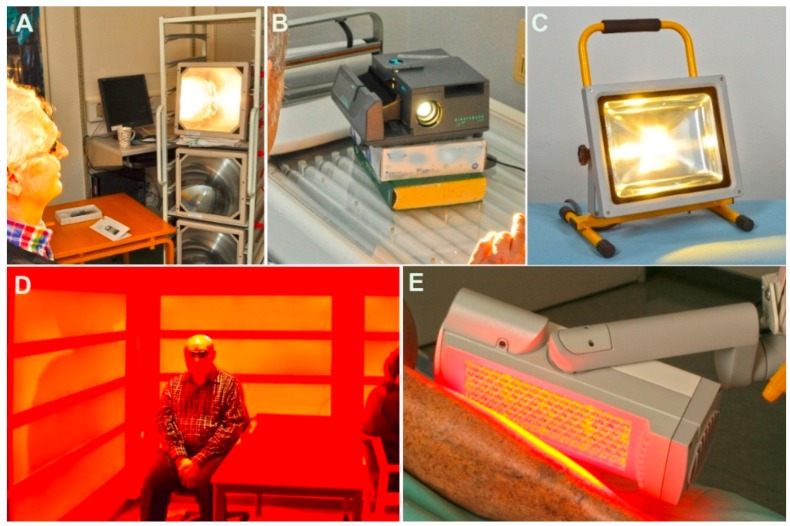
The five different light sources. (**A**) Overhead projector; (**B**) Slide projector; (**C**) White light-emitting diode (LED) lamp; (**D**) Red LED panel; (**E**) Red LED lamp.

**Figure 3:**
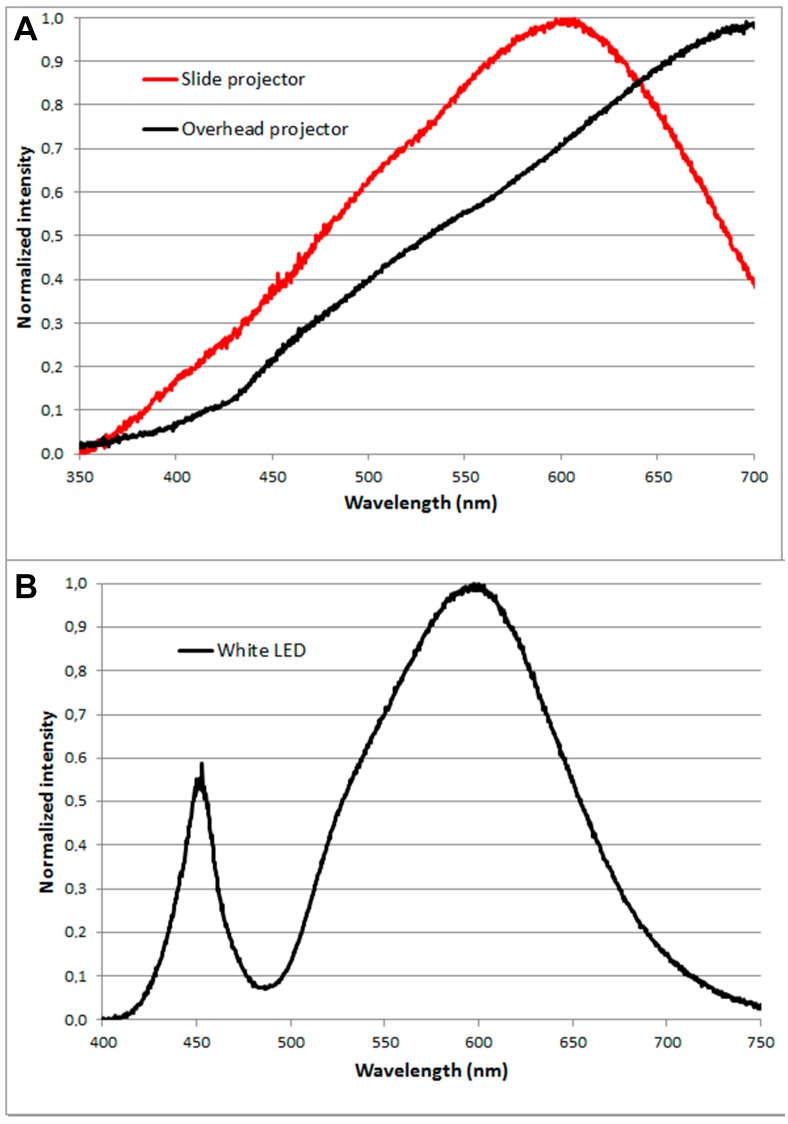

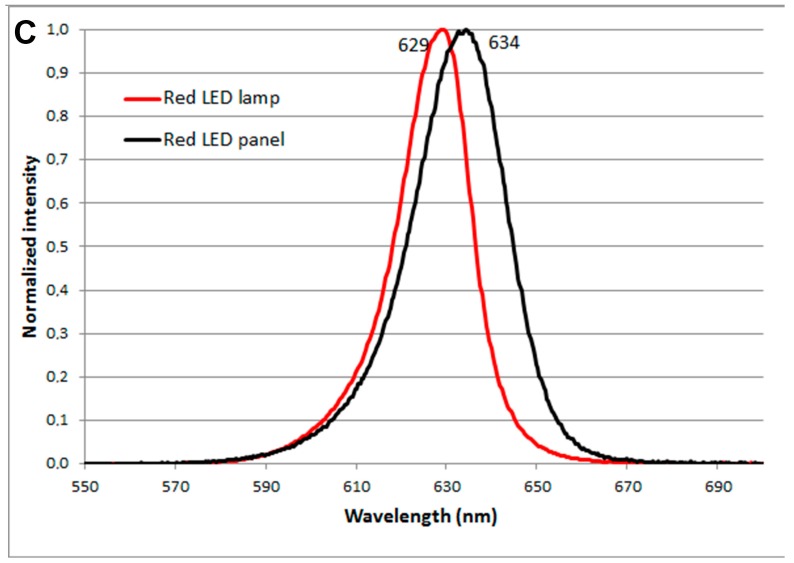
(**A**–**C**) Spectra of the five different light sources.

We investigated five different commercially available light sources. These were an overhead projector with dismantled mirror (Figure 2a), a slide projector (Figure 2b), a white LED lamp (Figure 2c), a red LED Panel (Figure 2d), and a red LED lamp used for conventional PDT (Figure 2e). In total, 15 different lamp types were measured, but only the five lamps with the most suitable light for the experiments were included. The results are shown in Table 1 and Figure 3.

The red LED lamp commonly used for conventional PDT has a peak intensity at 629 nm. For conventional PDT the lamp is set to give a total light dose of 37 J/cm^2^ in approximately 9 min. (at a distance of 8 cm). At a distance of 1 m the red LED lamp gave a fluence rate of 0.16 mW/cm^2^ (PpIX-weighted), which corresponds, to a fluence of 1.2 J/cm^2^ in two hours.

The overhead projector was the light source with the highest fluence rate of 1.14 mW/cm^2^ (PpIX-weighted) corresponding to 8.2 J/cm^2^. The slide projector resulted in a fluence rate of 0.17 mW/cm^2^ (PpIX-weighted) corresponding to 1.2 J/cm^2^ in two hours. The white LED lamp resulted in a fluence rate of 0.03 mW/cm^2^ (PpIX-weighted) corresponding to 0.2 J/cm^2^ in two hours and the red LED panel also resulted in a fluence rate of 0.03 mW/cm^2^ (PpIX-weighted) corresponding to 0.2 J/cm^2^ in two hours.

Five healthy volunteers were treated with a total of five different artificial “daylight” sources. After the skin was tape-stripped 10 times, it was incubated with MAL for 30 min. followed by illumination of the treatment fields with selected artificial “daylight” sources at varying distances for two hours.

The MAL-induced PpIX in the treatment fields illuminated with the artificial “daylight” was compared with the MAL-induced PpIX in control areas not illuminated.

The yields of fluorescence are given in Table 2. (Details of the experimental procedure are given in Section 3.) The skin temperature was stable after 20 min. of illumination and did not differ much between the light sources used (33–36 °C; Table 2). We measured the uniformity of the irradiation for five different places in each field (circle with a diameter of 5 cm) and the results were: Overhead projector 13,500 lux ± 2%, Slide projector 5000 lux ± 4%, White LED lamp 12,000 lux ± 7%, Red LED panel (4 panels of 30 cm × 120 cm) 2600 lux ± 4%, Red LED lamp 5000 lux ± 4%.

**Figure 4:**
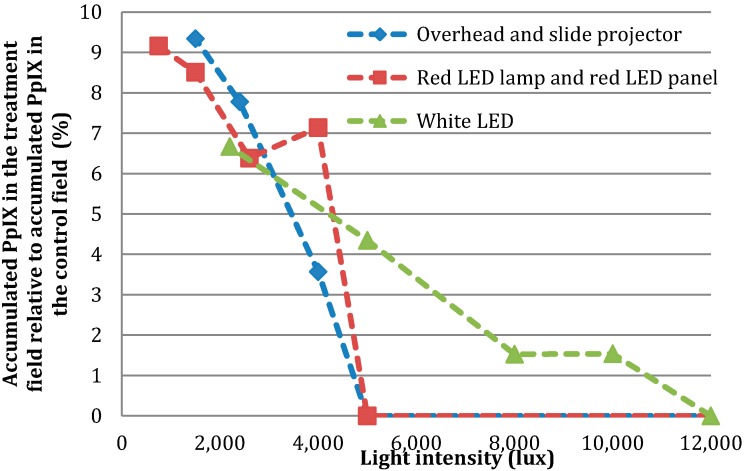
Percentage of unbleached PpIX by different light intensities.

We carried out illumination changing the distances between the light sources and the subject in order to determine the number of lux needed to achieve a complete photobleach. With the overhead projector it was not possible to use a lower number of lux than 13,500 because the light intensity is high. The overhead projector was included as we anticipated a high light fluence rate to be necessary. If we had wanted 5000 lux from the overhead projector, the distance from the subject to the light source would have had to be many meters. Regarding the white LED lamp 12,000 lux is needed for a complete photobleach in two hours, while it is 5000 lux for the slide projector and the red LED lamp. The highest light intensity the red LED panel can emit is 2600 lux and that is not enough to achieve a complete photobleach. In Figure 4 we have combined the data from the two halogen lamps and also the spectra from the two red LED light sources since their spectra are very similar despite the differences in intensity.

The post treatment erythema was not investigated systematically in this study but there did not seem to be a significant difference in post treatment erythema among the continuous light sources used. In contrast as expected, there was more post treatment erythema in the fields receiving conventional PDT compared to the fields that received continuous illumination.

### 2.2. Results from the Greenhouse

**Figure 5:**
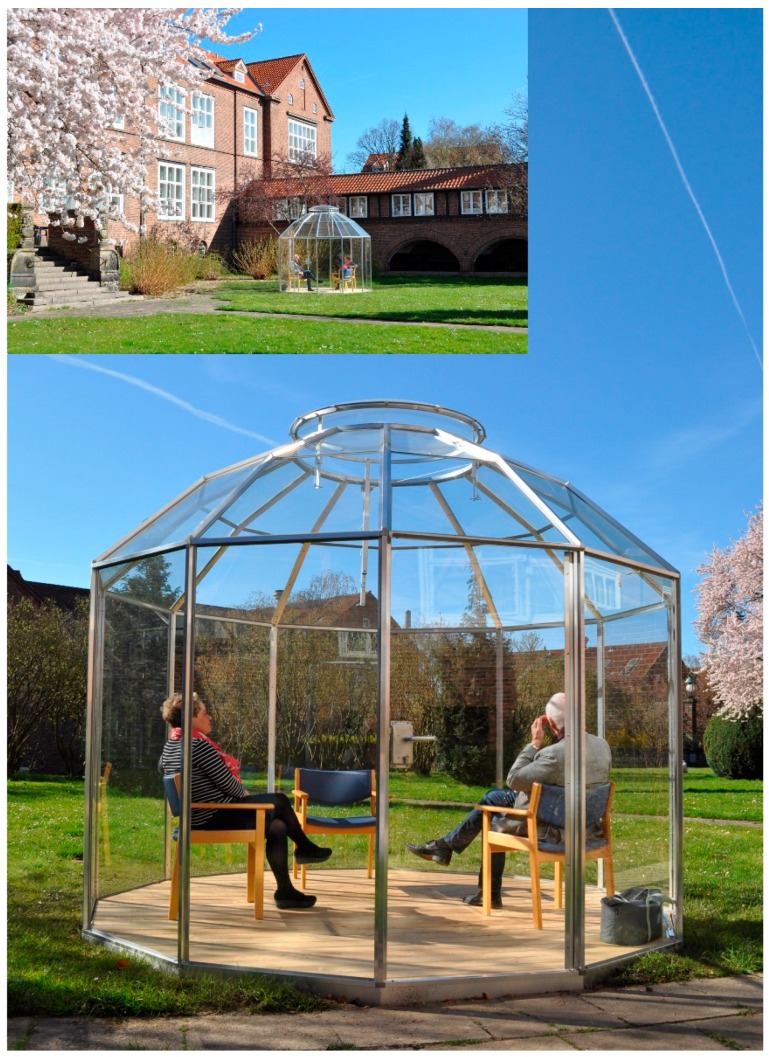
The greenhouse is constructed of one layer window glass with acrylic at the very top.

**Figure 6:**
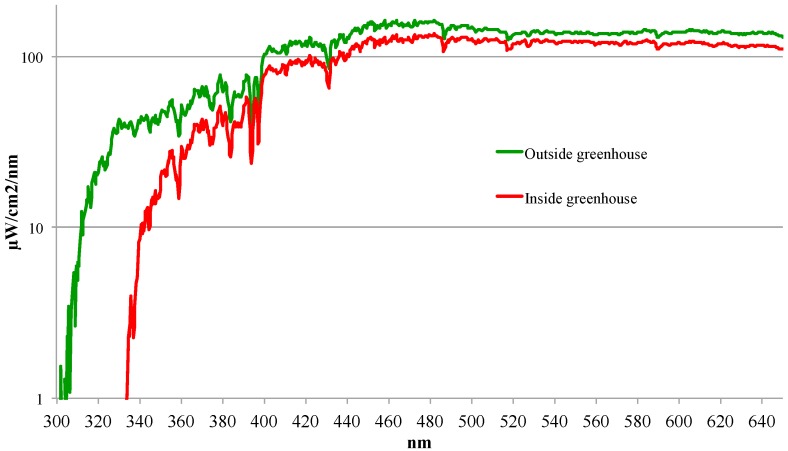
Spectra of daylight outside and inside the greenhouse–overcast (11 June 2015).

Daylight is the combination of direct and diffuse sunlight in the open air during the daytime. The greenhouse (Figure 5) makes it possible to perform daylight PDT even in harsh weather conditions. However, the ambient light intensity is only sufficient in April through October. We made the measurements inside and outside of the greenhouse during weather conditions with clear blue sky and heavy cloud (Table 3 and Figure 6). With clear blue sky the fluence rate of daylight outside the greenhouse was 6.07 mW/cm^2^ (PpIX-weighted) corresponding to 43.7 J/cm^2^ in two hours, while inside the greenhouse it was 4.58 mW/cm^2^ (PpIX-weighted) corresponding to 32.9 J/cm^2^ in two hours. Thus there was a difference of 1.5 mW/cm^2^, caused by the filter effect of the greenhouse glass. In overcast conditions the fluence rate of PpIX-weighted daylight was 1.69 mW/cm^2^ outside the greenhouse corresponding to 12.1 J/cm^2^ in two hours and 1.18 mW/cm^2^ inside the greenhouse corresponding to 8.5 J/cm^2^ in two hours resulting in a difference of 0.5 mW/cm^2^.

### 2.3 Discussion

A number of different light sources have tentatively been used for PDT including lasers, filtered xenon arc and metal halide lamps, fluorescent lamps and light-emitting diodes [1]. We have measured the absorption spectra from five different light sources, which might be usable for indoor “daylight” PDT. It is well known that continuous activation of PpIX by daylight during its development reduces treatment-related pain compared to that resulting from conventional PDT [1], but the weather does not always allow daylight PDT. Our measurements show that only the overhead projector is able to give the optimal fluence (PpIX-weighted) of 3.5–8 J/cm^2^ found in earlier studies of PDT treatment of actinic keratoses. Surprisingly, the results from the healthy volunteers show that it is possible to photobleach the developed PpIX using four of the five light sources even though the PpIX light dose is as low as 0.2 J/cm^2^. This means that the suggested threshold dose of 3.5–8 J/cm^2^ may be set somewhat lower [1]. We also measured the illuminance in lux because this measurement is easier for others to repeat as it can be done without special knowledge. We carried out illumination by changing the distances between the light sources to the subject in order to determine the number of lux needed to achieve a complete photobleach. The red LED panel is not usable for a complete photobleach since the intensity is not high enough. Regarding the white LED lamp 12,000 lux is needed for a complete photobleach in two hours while only 5000 lux is required for the other three light sources (Figure 4). A study by Wiegell *et al.* using Xenon H4 light bulbs showed that the minimal light intensity needed to prevent accumulation of PpIX which still resulted in an effective treatment of actinic keratoses was higher than 0.5–3.7 mW/cm^2^ or 1000–8000 lux [12]. Only the overhead projector was able to deliver fluence of this magnitude. Regardless of this, four of the five light sources were able to photobleach PpIX in healthy skin. Halogen light has previously been used as a light source for PDT [8,9]. A disadvantage of using halogen light is the production of heat, which can prove uncomfortable for the patients. A solution could be halogen light with less intensity. The other three light sources produce an insignificant amount of heat. However, in this study there was only a 3 °C increase in skin temperature after illumination with the halogen light.

The ideal alternative to daylight illumination for daylight PDT could be a combination of the different light sources. There may also be some patient types for whom some of the lamps are more appropriate than others. Thus, in our clinic we have an arrangement in which the slide projector is on a top shelf and is able to illuminate the patient with the help of a mirror. The advantage of lamps compared with daylight is the constant fluence rate directly on the lesion whereas daylight yields different fluence rates depending on the position of the patient. On the other hand the uniformity of irradiation from the lamps is an important issue. The light sources used in this study did not vary more than a Standard Deviation (SD) of 7%. Daylight PDT is especially useful for treatment of field cancerization. Therefore, ambient or diffuse light must be able to illuminate the entire treatment area. The studied light sources have the drawback that most of the light is direct and therefore the position of the patient relative to the light source will have a large impact on PpIX photobleaching. Also, topical alternative therapies are available and it will normally not be a major problem or a great risk to postpone daylight PDT for actinic keratosis until spring or summer. However, in our experience daylight PDT is a very tolerable treatment with good compliance. It is easy for elderly patients to have one time daylight PDT compared with self-administration of topical formulations for a longer time period. We could postpone the patients until spring or summer but we are getting more and more patients with AKs so it is useful to be able to perform daylight PDT all year round.

These findings open perspectives for optimized PDT treatment, and future studies are needed to clarify whether indoor daylight PDT using the light sources described in this paper is as effective a treatment as conventional PDT in patients with actinic keratoses.

Our measurements in the greenhouse show that 25%–30% of the fluence is filtered by the greenhouse glass. There is an even suppression of the light intensity across the wavelengths in the PpIX spectrum. However, as expected there is suppression of the UVB-radiation by the glass. Measurements showed that it was possible to receive 9.4 Standard Erythema Dose (SED)/per hour out in the open on the day of clear blue sky compared with 0.8 SED inside the greenhouse. An average citizen in Denmark can receive 3–4 SED before developing erythema. Accordingly, sun protection for patients using the greenhouse becomes less important. 

The greenhouse can be used for daylight PDT during the summer because the light intensity is high. In periods of lower light intensity, e.g. October-March in Denmark there is a risk of not reaching the treatment threshold.

## 3. Experimental Section

### 3.1. Light Sources

The spectra of the lamps were recorded at different distances using a spectroradiometer (Jaz, Ocean Optics, Florida, FL, USA). Illuminance was measured with a lux meter, model E2 (Hagner, Solna, Sweden). We used the following five light sources: (1) a slide projector (Kindermann GmbH, Eibelstadt, Germany) model Silent 2500, which is mounted with a 250-W halogen light bulb. Light from the light bulb shines through the empty slide holder, condenser lenses and the projection lens. A concave mirror behind the lamp also helps to direct the light. The slide projector has a heat filter, which makes the spectrum above 630 nm different from that of the overhead projector; (2) an overhead projector (Medium GmbH, Düsseldorf, Germany), model OHP 536. It is mounted with a 400-W halogen light bulb. An overhead projector is similar to the slide projector. Normally, after the light leaves the projection lens, an angled plane mirror reflects and reverses the image so it appears right-side up on a vertical projection screen (mirror dismantled). The light from the lamp is directed towards the projection lens by a plastic lens of the Fresnel type placed beneath the glass plate on top of which the transparency is placed; (3) a white LED lamp (Nor-tec, Kolding, Denmark) model 74,643 with a 50-W LED emitter; (4) an LED panel with diffuse light consisting of four panels of 30 cm × 120 cm (Cada Light, Shenzhen, China) model BL-PL 1203-18W, in which each panel is 18 W; (5) the red LED lamp often used for conventional PDT (Photocure ASA, Oslo, Norway) model Aktilite CL 128 140 W.

### 3.2. Exploratory Investigation

Five healthy volunteers of Scandinavian origin were included in the study (mean age 46, range 27–72). On each volunteer three circular skin fields, two fields on one arm and one field on the other arm, with a diameter of 5 cm (19.6 cm^2^) were identified. The borders of each field were marked with a black non-fluorescent marker using a template. One of the fields on each arm was chosen for treatment. The remaining field was chosen as a control field. Both inner and outer arm were used. The control field was always placed on the same site near by the treatment field. In order to imitate skin lesions all fields were initially tape stripped 10 times (Lyreco Budget clear sticky tape, Marly, France). An excess of MAL 16% cream (Metvix^®^, Galderma, Lausanne, Switzerland) was applied on all fields. These were covered with a light-impermeable occlusive dressing. After 30 min. the dressing was removed from the two treatment fields, which were then illuminated for two hours with different lamps.

Immediately after the two hours the light-impermeable dressing was removed from the control field and excess cream wiped off. All the fields were then illuminated with the red LED lamp with a total light dose of 37 J/cm^2^ over 8 min—the same illumination as used during conventional PDT. PpIX fluorescence yields were measured in all three fields just before and after this illumination.

PpIX fluorescence was measured non-invasively using a handheld fluorescence photometer (FluoDerm, DiaMedico, Gentofte, Denmark) [13]. The photometer illuminates a skin area with a diameter of 4 cm with blue light (400–420 nm light-emitting diodes) matching the Soret band of PpIX at 407 nm. The corresponding red PpIX fluorescence intensity at 633 nm is also detected. Measurements were performed in arbitrary Fluoderm Units. For each field the difference between the two fluorescence measurements was determined. This figure expressed how much PpIX had been accumulated over the preceding 180 min, adjusted for the autofluorescence of the skin. Only the difference in fluorescence yield was analyzed and presented. The skin temperature was measured with a infrared thermometer RS 1327 (RS Components Ltd., Northants, UK).

### 3.3. Greenhouse

The greenhouse is constructed of one layer window glass with acrylic at the very top (Figure 5). A UV dosimeter [14] was continuously measuring ambient UV while the spectroradiometric measurements were performed outside and inside the greenhouse to verify that the ambient light did not change during the measurements. The timespan between the measurements was less than 10 min, so it can be assumed that the solar spectrum did not change. The effect of the greenhouse glass was calculated by comparing the two spectroradiometric measurements.

## 4. Conclusions

We found that among the lamps described here, halogen light (overhead and slide projector) was the best “indoor daylight lamp” for PDT treatment. Four of the five light sources were able to photobleach the PpIX produced in the skin completely. Furthermore, we found that the greenhouse is suitable for daylight PDT when the weather is cold, windy or wet since it filters out only 25%–30% of the daylight. Depending on the geographical location, the greenhouse can be used in the months when the intensity of the daylight is high enough but the weather is too cold for patients to be outside (e.g., between April and October in Denmark).

## Figures and Tables

**Table 1:** Fluence rate (PpIX-weighted full spectrum), fluence (PpIX light doses), intensity of visible light (lux) and how many lux are needed for a complete photobleaching in two hours using the five different light sources. All numbers in the table are for the distance of 1 meter except for the last column (see distances for the last column in Table 2). The visible light intensity is shown for simplicity because it is inexpensive and very easy to measure without prior knowledge.

Lamp Type	Fluence Rate–Full Spectrum (mW/cm^2^)	Fluence Rate–Full Spectrum–PpIX-Weighted (mW/cm^2^)	Fluence-PpIX Light Dose for 2 h (J/cm^2^)	Visible Light Irradiance (lux)	Irradiance Giving 100% Effect on PpIX (lux)
Overhead projector 400 W	18.12	1.14	8.2	35,000	5000
Slide projector 250 W	2.04	0.17	1.2	5800	5000
White LED lamp 50 W	0.53	0.03	0.2	1630	12,000
Red LED panel 18 W	0.89	0.03	0.2	1420	5000
Red LED lamp Aktilite 140 W	5.20	0.16	1.2	17,840	5000

**Table 2:** Accumulation of PpIX during two hours of illumination with the different artificial “daylight” sources. Numbers 1 and 2 are both halogen light, while 4 and 5 are both red LED light (Figure 3). The skin temperature was 33 °C before illumination in all volunteers. The skin temperature given in Table 2 was stable after 20 min of illumination.

	Light Source	Healthy Volunteer	Distance to the Lamp (m)	Visible Light Intensity (lux)	Skin Temperature (°C)	Unbleached PpIX (%)
1	Overhead projector	A	1	38,000	-	0
	400 W	B	1.5	13,600	-	0
		A	1.5	13,500	36	0
2	Slide projector	A	1.1	5000	-	0
	250 W	B	1.1	5000	33	0
		B	1.3	4000	-	4
		C	1.5	2400	-	8
		D	2	1500	-	9
3	White LED lamp	D	0.3	12,000	35	0
	50 W	B	0.4	10,000	-	2
		D	0.4	8000	-	2
		B	0.6	5000	-	4
		C	1	2200	-	7
4	Red LED panel (4 panels of 30cm × 120 cm)	C	0.1	2600	33	6
	18 W each panel	C	0.4	1500	-	9
		D	1.2	750	-	9
5	Red LED lamp	A	1	17,300	-	0
	Aktilite	B	1	15,000	-	0
	140 W	B	1.5	5000	33	0
		B	1.9	4000	-	7
	Greenhouse	E	-	8500	-	0

**Table 3:** PpIX-weighted full spectrum and PpIX light doses in two hours of daylight outside and inside the greenhouse during different weather conditions.

Lamp Type	Fluence Rate–Full Spectrum PpIX Weighted (mW/cm^2^)	Fluence-PpIX Light Dose in 2 h (J/cm^2^)
Greenhouse—outside Clear blue sky	6.07	43.7
Greenhouse—inside Clear blue sky	4.58	32.9
Greenhouse—inside Heavily overcast	1.18	8.5
Greenhouse—outside Heavily overcast	1.69	12.1
